# The Venom of the Ectoparasitoid Wasp *Pachycrepoideus vindemiae* (Hymenoptera: Pteromalidae) Induces Apoptosis of *Drosophila melanogaster* Hemocytes

**DOI:** 10.3390/insects11060363

**Published:** 2020-06-11

**Authors:** Bin Wan, Lei Yang, Jiao Zhang, Liming Qiu, Qi Fang, Hongwei Yao, Marylène Poirié, Jean-Luc Gatti, Gongyin Ye

**Affiliations:** 1 State Key Laboratory of Rice Biology & Ministry of Agriculture and Rural Affairs Key Lab of Molecular Biology of Crop Pathogens and Insects, Institute of Insect Sciences, Zhejiang University, Hangzhou 310058, China; wan-bin1234@hotmail.com (B.W.); yanglei@zju.edu.cn (L.Y.); zhangjiao@zju.edu.cn (J.Z.); 21816179@zju.edu.cn (L.Q.); fangqi@zju.edu.cn (Q.F.); hwyao@zju.edu.cn (H.Y.); 2 Institut Sophia Agrobiotec h (ISA), Institut National de la Recherche Agronomique (INRA), Centre National de la Recherche Scientifique (CNRS), Université Côte d’Azur, 06903 Sophia Antipolis, France; marylene.poirie@inrae.fr (M.P.); jean-luc.gatti@inrae.fr (J.-L.G.)

**Keywords:** *Pachycrepoideus vindemiae*, *Drosophila melanogaster*, ectoparasitoid, venom, apoptosis, cell immunity

## Abstract

The pupal ectoparasitoid *Pachycrepoideus vindemiae* injects venom into its fly hosts prior to oviposition. We have shown that this venom causes immune suppression in *Drosophila melanogaster* pupa but the mechanism involved remained unclear. Here, we show using transgenic *D. melanogaster* with fluorescent hemocytes that the in vivo number of plasmatocytes and lamellocytes decreases after envenomation while it has a limited effect on crystal cells. After in vitro incubation with venom, the cytoskeleton of plasmatocytes underwent rearrangement with actin aggregation around the internal vacuoles, which increased with incubation time and venom concentration. The venom also decreased the lamellocytes adhesion capacity and induced nucleus fragmentation. Electron microscopy observation revealed that the shape of the nucleus and mitochondria became irregular after in vivo incubation with venom and confirmed the increased vacuolization with the formation of autophagosomes-like structures. Almost all venom-treated hemocytes became positive for TUNEL assays, indicating massive induced apoptosis. In support, the caspase inhibitor Z-VAD-FMK attenuated the venom-induced morphological changes suggesting an involvement of caspases. Our data indicate that *P. vindemiae* venom inhibits *D. melanogaster* host immunity by inducing strong apoptosis in hemocytes. These assays will help identify the individual venom component(s) responsible and the precise mechanism(s)/pathway(s) involved.

## 1. Introduction

Female parasitoid wasps oviposit in or on a host, their eggs developing using the host as a food source and the host eventually dying as a result of the parasitoid’s development. Parasitoid wasps are natural enemies of many insects, including agricultural pests and are therefore used as biological control agents. Most of these wasps inject venom into their host prior or during oviposition, which contains various factors necessary to regulate the host physiology for the successful development of the parasitoid egg. For endoparasitoids that develop inside their host, maternal factors mainly protect eggs from the host immune response, encapsulation, that is, the covering of the egg by the immune cells and the melanization leading to its death [1]. In contrast, ectoparasitoids that develop outside the host inject maternal factors that mainly paralyze or block the host development and, in some cases, also affect the host immune system [2,3,4,5].

The venom factors of bot h endoparasitoids and ectoparasitoids comprise various active components, from bioactive compounds to proteins and particles such as polydnavirus (PDVs), virus like-particles (VLPs) and venosomes [6,7,8,9]. The role of bioactive compounds and proteins in the regulation of immune responses of host cells is still poorly understood [5,10] unlike that of venom particles which has been largely explored. The injection of purified VLPs from *Meteorus pulchricornis* into the larva of its host moth *Pseudaletia separata* led to rapid disassembly of the leading edge of filopodia and lamellipodia into the host granular cells, a kind of phagocytic cells and the parasitism as well as the injection of VLPs induced apoptosis of the host hemocytes [11,12]. Likewise, both the injection of PDVs from *Microplitis demolitor* into the hemocoele of *Pseudoplusia includens* larvae and the in vitro incubation with hemocytes induced apoptosis and chromatin condensation in the granular cells of the host [13]. It also appeared that the injection of purified venosomes from *Leptopilina* species, endoparasitoids of *Drosophila*, selectively induced shape changes/death of the lamellocytes, the hemocyte type specialized in the encapsulation of parasitoid eggs and therefore contributed to the success of parasitism [14,15,16].

The venom of *Nasonia vitripennis*—a model species for ectoparasitoids that lay eggs on the host—also produces a variety of toxic effects including arresting the development of the host and lowering metabolism and immunity [17,18]. A subsequent study demonstrated that the venom of *N. vitripennis* induces rapid apoptosis of cultured cells and alters their mitochondrial transmembrane potential [19]. A more recent transcriptomic study showed that this venom induces the differential expression of a battery of genes related to various functions such as immunity, apoptosis, stress response and metabolism in the pupal hemocytes of the host *Musca domestica* [20]. Altogether, it can be concluded that the venom components of bot h endoparasitoids and ectoparasitoids play multiple roles, notably inhibiting the host immune responses, in particular by disturbing the functions of host hemocytes.

*Pachycrepoideus vindemiae* (Hymenoptera: Pteromalidae) is a pupal ectoparasitoid phylogenetically related to *N. vitripennis*. It has a wide-range of dipteran fly hosts, including Drosophilidae, Anthomyiidae, Calliphoridae, Muscidae and Sarcophagidae [21]. *P. vindemiae* can superparasitize already infected hosts but ultimately only one parasitoid will survive and emerge as an adult, confirming its solitary status [22]. Ectoparasitoids have been much less studied than endoparasitoids and *P. vindemiae-D. melanogaster* is becoming a model to analyze the effect of virulence factors of such wasps using this well described fly model. The immunity of *D. melanogaster* relies on well-described humoral and cellular responses [23,24]. The larval immune response involves three main types of hemocytes each with specific functions: the plasmatocytes involved in phagocytosis, the crystal cells in the melanization and the lamellocytes in the process of encapsulating parasitoids [24]. Plasmatocytes and crystal cells are produced constitutively while the number of lamellocytes increase before pupation or in response to oviposition of parasitic wasps [24,25,26,27]. 

Our recent work has demonstrated that the venom of *P. vindemiae* is composed of a large number of proteins [28] responsible for manipulating the host physiology, including the immune system [2], although the mechanisms involved are largely unknown. In the present study, we monitored the dynamic changes in the number of plasmatocytes, lamellocytes and crystal cells after parasitism by *P. vindemiae* using *D. melanogaster* transgenic lines with hemocytes expressing a specific GFP (Green Fluorescent Protein) tag. Envenomation by *P. vindemiae* resulted in a significant decrease in host hemocyte numbers. In vitro studies with host hemocytes have demonstrated that the venom induces a rearrangement of the cell cytoskeleton, fragmentation of the nucleus and apoptosis. This was confirmed by transmission electron microscopy. These data provide a better understanding of the functions of the ectoparasitoid venom and the various in vivo and in vitro assays performed will be tools in the future to analyze which individual venom component(s) induce cell apoptosis and characterize the mechanism(s) involved.

## 2. Materials and Methods

### 2.1. Insect Rearing

The colony of *P. vindemiae* was kindly provided by Prof. Yongyue Lu (South China Agricultural University, Guangzhou, China) and was reared at 25 °C on pupae of the *D. melanogaster* strain W^1118^. After emergence, the adult wasps were kept in glass containers and fed with a 20% (*v/v*) honey solution at 25 °C. The wasps used for experiments were mated females aged 3–8 days.

The *D. melanogaster* stocks were obtained from the Bloomington Stock Center (Indiana University, Bloomington, IL, USA): Hml-GFP (stock ID: 30140), Atilla-GFP (stock ID: 23540), Lozenge-GFP (stock ID: 6313) and hop^Tum-l^ (stock ID: 8492). All *Drosophila* stocks were reared on standard medium at 25 °C with 60 ± 5% relative humidity and 16 h:8 h (light: dark) photoperiod [29].

### 2.2. Parasitism Assay

Fifty *D. melanogaster* pupae were collected 4 h after pupation and transferred to tube for 48 h before being parasitized for 3 h with eight *P. vindemiae* females. Subsequently, the wasp eggs were removed and the pupae were kept at 25 °C for 2 h, 10 h and 36 h until use. Unparasitized *D. melanogaster* pupae of the same age were used as control.

### 2.3. Venom Collection 

Mated female wasps were anaesthetized at 4 °C for 10 min and then dissected in sterile PBS (Phosphate Buffer Solution) on an ice plate under a stereoscope (JSZ6, Nanjing Jiangnan Novel Optics Co., Nanjing, China). The venom reservoir was separated and washed in PBS, transferred in a 1.5 mL tube and centrifuged at 1000 g for 10 min, then the supernatant was transferred into a new 1.5 mL tube and used for the studies. 1 VRE represents the contain of one venom reservoir collected in 1 µL of PBS.

### 2.4. Whole Pupae Fluorescence Microscopy

Whole *Drosophila* pupae with fluorescent hemocytes (parasitized or unparasitized, at least ten for each experiment) were observed after carefully removing the pupal envelop with tweezers and photographed using the GFP fluorescence channel of the microscope (Nikon AZ100M; Nikon, Tokyo, Japan). Bright fluorescent dots considered to be hemocytes were manually counted using the Zen lite software (Zeiss, Iena, Germany). Due to the technical approach chosen to visualize the whole pupa, only cells or clusters of cells close to the pupal surface could be observed. The number of reported spots is therefore representative of the total number of cells considered. While this method has been previously used to visualized and estimate the fluorescent hemocytes number in *Drosophila* larvae [30], the parasitism-induced change in the number of pupal hemocytes after 10 h was also assessed by a direct counting method on a hemocytometer (see details in Figure S1). Since the results of bot h methods were clearly correlated and the microscopic approach on whole fluorescent pupae was less time consuming, we used it for all experiments.

### 2.5. Hemocytes Collection and Immunocytochemical Staining

The hemocytes were obtained from third instar hop^Tum^^-l^ larvae which constitutively display an over-proliferation of hemocytes, notably lamellocytes and plasmatocytes [31,32]. Briefly, three hop^Tum^^-l^ larvae were carefully washed three times in 10 mM PBS (pH 7.4). The larvae were bled by carefully tearing the anterior cuticle of larvae with forceps on a 25 µL drop of PBS deposited on a round glass coverslip (diameter = 14 mm). The coverslip was transferred to a 12-well culture plate placed in a wet chamber. After leaving the hemocytes adhering for 15 min, they were incubated with the indicated venom reservoirs equivalent (from 0 to 6 VRE) and for the indicated time (1 h or 3 h). Thereafter, we washed the hemocytes twice with PBS and fixed them with a 4% paraformaldehyde solution (Sangon Biotech, Shanghai, China) for 15 min at 25 °C, before washing them three times with PBS for 30 min. The excess of liquid was carefully drained and the samples were permeabilized for 5 min with 0.1% Triton X-100 followed by three washes with PBS.

*For counting the fragmented nuclei*, the hemocytes adhered for 1 h and were then incubated with 3 VRE for 1 h. After fixation and permeabilization, the cell actin cytoskeleton was stained with phalloidin iFluor 488 (1:1000; Abcam, Cambridge, UK) diluted in PBS-0.3% BSA for 1 h. Then, the hemocytes were washed twice with PBS for 20 min and mounted with an antifading medium containing DAPI (SlowFade^TM^ Gold; Life Technologies, Carlsbad, CA, USA). The samples were observed with an inverted fluorescent microscope (Nikon eclipse TS-100; Nikon, Japan) and pictures taken with a fluorescent sensitive camera (Axiocam 503 mono; Zeiss). The samples analyzed were obtained from at least three separate experiments (approximately one hundred cells counted per slide).

*For TUNEL assay*, washed permeabilized hemocytes were treated as described by the manufacturer (TUNEL BrightGreen Apoptosis Detection Kit; Vazyme, Nanjing, China). Briefly, to fluorescently label the free hydroxyl terminus of DNA with FITC-12-dUTP, the cells were incubated with 100 µL of Equilibration Buffer (EB) for 20 min and then with Terminal deoxynucleotidyl Transferase (TdT) (34 µL ddH_2_O, 10 µL EB5x, 5 µL BrightGreen Labeling Mix and 1 µL TdT Enzyme) at 37 °C for 60 min. After three times washes with PBS, hemocytes were mounted with an antifading medium containing DAPI and observed as above. As a positive control, permeabilized hemocytes were incubated with DNAse (10 U/mL during 15 min; from Vazyme) before labeling. Samples from at least three separate experiments were analyzed (approximately one hundred cells counted per slide).

*For caspase inhibition*, hemocytes were preincubated 15 min with PBS or the irreversible pan-caspase inhibitor Z-VAD-FMK (150 µM, Selleck, WA, USA) before 1 h incubation with 4.5 VRE. Then, the hemocytes were fixed, permeabilized, stained and observed as above. Samples from at least three separate experiments were analyzed.

### 2.6. Electron Microscopy

The hemolymph from 100 hop^Tum-l^ larvae was collected in 150 μL of PBS and then transferred to a 1.5 mL tube and either 30 μL PBS or 30 VRE was added. After 3 h of incubation at 25 °C, the hemocytes were centrifuged at 2000 g for 10 min and the cell pellets were fixed with 5% glutaraldehyde for 24 h at 4 °C. The cell pellet was then embedded in 2% agarose diluted in PBS. After three washes with PBS, the cells were treated with 2% osmium tetroxide for 2 h, rinsed in PBS and rapidly dehydrated by a step series of ethanol (from 30% to 100%) for 10 min each, followed by a final 20 min dehydration in acetone. Then, the samples were incubated 1 h in 1:1 mixture of absolute acetone and the final Spurr resin mixture (SPI-Chem Low Viscosity; SPI, West Chester, PA, USA), transferred to a 1:3 mixture of absolute acetone and the resin mixture for 3 h and then to a final pure Spurr resin mixture overnight. The block was then transferred into microtubes and polymerized for 12 h at 70 °C. To visualize the hemocytes, we first cut thick sections (1 μm; LKB 11800 PYPAMITOME, LKB-Produkter AB, Stockholm, Sweden) and collected the best samples for the observation. Thin sections (90 nm) were then done (LEICA EM UC7; Leica microsystem, Germany) and contrasted using uranyl acetate and lead citrate before observation and imaging by a 80 kV Hitachi H-7650 electron microscope (Hitachi, Tokyo, Japan).

### 2.7. Data Analysis

To distinguish the different types of hemocytes, these cells were stained with phalloidin and then their area was measured using image processing software (Image J 1.8.0; NIH, Bethesda, MD, USA). Data from two groups were analyzed by a two-tailed unpaired Student’s t-test. All statistical analyses and figures were plotted using GraphPad Prism 7.0 (GraphPad, San Diego, CA, USA). All values are represented on average by three repetitions with standard deviation. *p*-values less than or equal to 0.05 were considered significant.

## 3. Results

### 3.1. Effects of Parasitism on Host Hemocytes

To monitor dynamic changes due to envenomation by *P. vindemiae*, we parasitized the pupae and removed the parasitoid egg immediately afterwards to prevent the developing larva from injecting effectors or affecting pupal metabolism. The effect of the venom on the number of hemocytes was followed using transgenic *D. melanogaster* lines expressing the GFP marker for each type of hemocyte. Since pupae did not have a clear hemolymph which could be collected like larvae or adult fly, we used an indirect microscopy method which allows a good estimate of the total number of the hemocytes considered (see Figure S1 and Reference [30]).

**Figure 1:**
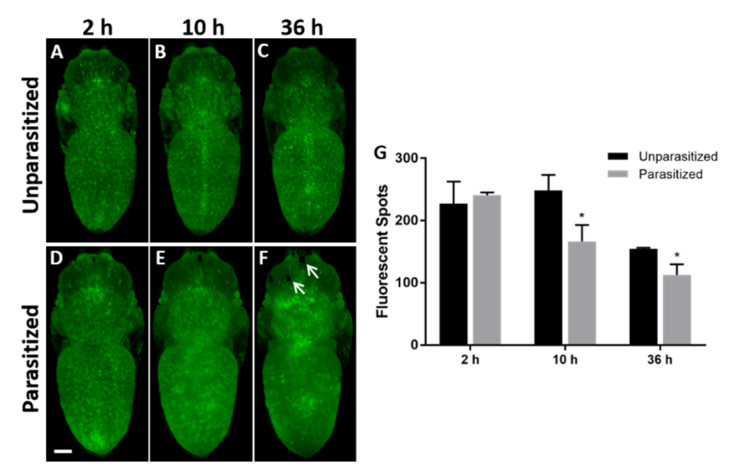
**Effects of *P. vindemiae* parasitism on the number of plasmatocytes (Hml > GFP).** In whole *Drosophila* Hml > GFP (Green Fluorescent Protein) pupae, the plasmatocytes were observed as bright green fluorescent spots 2 h, 10 h and 36 h in unparasitized controls (**A**–**C**) and after parasitism (**D**–**F**). The arrows show the black spots formed at the oviposition sites (**F**). (**G**) Quantification based on the number of fluorescent spots shown as the mean ± S.D. for each time and condition; *: *p* < 0.05 (*n* = 10 pupae, from three separate experiments). Bar = 250 μm.

*Plasmatocytes*. Hml-GFP is expressed in plasmatocytes (see Figure S2) and crystal cells but not in lamellocytes [33,34]. Since plasmatocytes represent >90% of the circulating hemocytes in third instar larvae, the fluorescent spots in Hml-GFP pupae (Figure 1) were considered to be plasmatocytes. The pictures show that their overall distribution remained similar between the parasitized (Figure 1A–C) and the unparasitized (Figure 1D–F) pupae during the 36 h observation period, except for those which accumulate around the dorsal vessel that did not form after parasitism. When counting the number of fluorescent spots under normal conditions (Figure 1G), we observed a stable number of pupal plasmatocytes during the first 10 h, then a sharp reduction at 36 h. Two hours after parasitism, the number of plasmatocytes was identical to that of unparasitized pupae whereas it was significantly reduced compared to the control after 10 h and 36 h (loss of 33% and 27%, respectively; Figure 1G). Therefore, the envenomation led to an earlier decrease in the number of pupal plasmatocytes together with a higher loss at 36 h.

*Crystal cells*. The transcription factor *lozenge* (Lz) being classically used as a marker for crystal cells [35,36], a Lozenge-GFP line of *Drosophila* was used to visualize their becoming after envenomation. Very few fluorescent dots were observed in both control and parasitized pupae, as expected since the crystal cells represent less than 5% of the larval hemocytes, therefore around a hundred cells per larva (Figure S3A–C and Figure S3D–F, respectively). As for plasmatocytes, the number of crystal cells decreased over time during pupation (Figure S3G). The parasitism caused a significant decrease of 31% in the number of fluorescence spots compared to the control but only 2 h after parasitism (Figure S3G).

**Figure 2:**
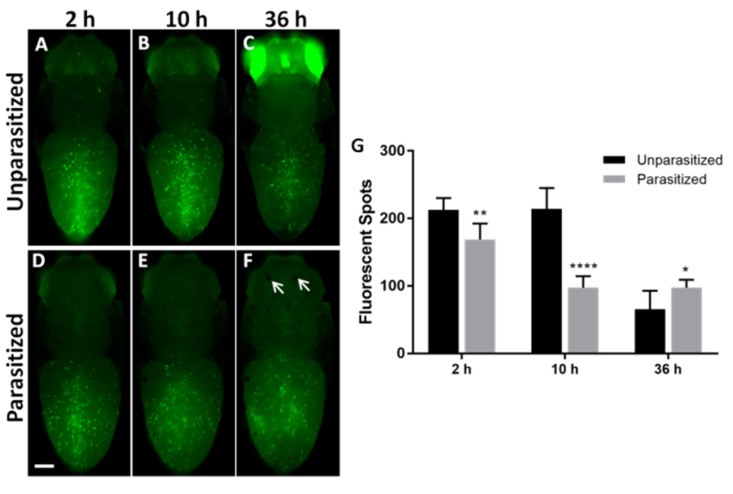
**Effects of *P. vindemiae* parasitism on the number of lamellocytes (Atilla > GFP).** Atilla > GFP lamellocytes were visualized as fluorescent green spots in pupal *Drosophila* at 2 h, 10 h and 36 h in unparasitized controls (**A**–**C**) and after parasitism (**D**–**F**). The arrows in (**F**) show the oviposition sites. (**G**) Quantification based on the number of fluorescent spots shown as the mean ± S.D. for each time and condition; *: *p* < 0.05, **: *p* < 0.01 and ****: *p* < 0.0001 (*n* = 10, from three separate experiments). Bar = 250 μm.

*Lamellocytes*. Atilla being a membrane protein of the lamellocytes, the *Drosophila* Atilla-GFP line has fluorescent lamellocytes [37] (see Figure S4). As with plasmatocytes, the pictures show an almost similar distribution of lamellocytes in unparasitized and parasitized pupae (Figure 2). The number of lamellocytes remained stable in the non-parasitized larvae until 10 h, then decreased sharply (Figure 2G). The decrease in number of the lamellocytes occurred earlier in parasitized pupae compared to the control, with a significant reduction of 21% and 55% after 2 h and 10 h, respectively. However, at 36 h, the parasitized pupae retained a higher number of lamellocytes than the control ones (Figure 2G). 

Interestingly, we observed that the different drivers induced also GFP in different tissues of the non-parasitized pupae such as dorsal vessel (Figure 1) or the eyes (Figure 2 and Figure S3), an induction strongly inhibited after parasitism. This confirmed a wider toxic effect of the venom on the different pupal tissues and therefore on the fly development.

### 3.2. Effects of the Venom on the Hemocyte Morphology

**Figure 3:**
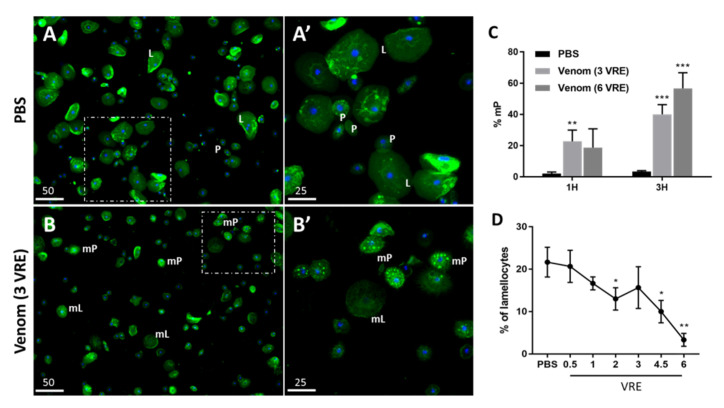
**Effect of crude venom on the morphology of host hemocytes *in vitro*.** Hemocytes collected from 3^rd^ stage hop^Tum-l^ larvae were incubated with PBS (**A**) or venom (**B**) for 1 h and stained for actin with green phalloidin and nucleus with DAPI (blue). (**A’**) and (**B’**) are enlargement of the dashed area indicated in (**A**) and (**B**) respectively. P, normal plasmatocytes; mP, modified plasmatocytes with aggregated actin spots in the cytoplasm. L, normal lamellocytes; mL, modified lamellocytes with damaged nucleus. (**C**) Estimation of the percentage of modified plasmatocytes, mP, after 1 h and 3 h incubation with PBS or 3 or 6VRE treatment. (**D**) Quantification of the number of lamellocytes after 1 h incubation with PBS and different VRE. Mean ± S.D. (*n* = 3); *: *p* < 0.05, **: *p* < 0.01 and ***: *p* < 0.001. Bar = 25 µm and 50 µm.

The hemocytes, obtained from 3rd stage mutant hop^Tum-l^ larvae which constitutively express a large number of lamellocytes [31], were let adhere to the glass and incubated either with PBS as a control or with venom. Figure 3A shows adherent hemocytes in PBS stained for actin allowing the identification of plasmatocytes, small round cells of about 10 µm and lamellocytes, large flat round cells of about 30–40 µm (Figure 3A,A’). In PBS, all cells had a blue nucleus clearly visible by DAPI staining. In contrast, after 1 h incubation with 3 reservoir equivalents (3 VRE), the plasmatocytes were modified (mP; Figure 3B,B’) with extended pseudopods and bright intracellular spots due to actin aggregations in the cytoplasm. After 3 h incubation (Figure S5), the number of aggregated actin spots in the plasmatocytes increased from 22.7% to 40% (Figure 3C). Incubation with 6VRE for 1 h and 3 h further increased the number of aggregated actin spots in the plasmatocytes by 18.7% (non-significant) and 56.7%, respectively (Figure 3C). In a dose effect study, the venom threshold affecting plasmatocytes has been set at 3VRE (Table S1).

**Figure 4:**
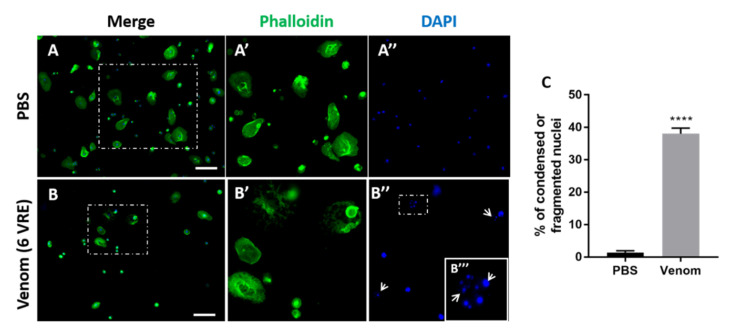
**Detection of condensed or fragmented nuclei in hemocytes**. Hemocytes collected from 3rd stage hop^Tum-l^ larvae were incubated for 1 h with PBS (Phosphate Buffer Solution) (**A**) or 6VRE (**B**) and labeled for actin (green) and nucleus (blue). (**A’**–**A’’**), (**B’**–**B’’**), (**B’’’**) are enlargement of the area indicated in (**A**), (**B**), (**B’’**) respectively, showing actin or nucleus labeling. Arrows in (**B’’**), (**B’’’**) show condensed or fragmented nuclei of lamellocytes. (**C**) Percentage of lamellocytes having a condensed or fragmented nucleus. Mean ± S.D. (*n* = 3); ****: *p* < 0.0001. In (**A**), (**A’**), (**A’’**), (**B**), bar = 50 µm. In (**B’**), (**B’’**), Bar = 15 µm.

For the lamellocytes, we observed after treatment with venom a weaker actin labeling and a shape which became irregular with an unclear cell edge (Figure 3A’–B’). In addition, increasing the venom concentration from 0 to 6VRE gradually decreased the number of lamellocytes stuck on the slide from 22% to 4% (Figure 3D) suggesting a strong effect on cell adhesion or viability. Indeed, when the adherent hemocytes were incubated with the 6VRE for 1 h (Figure 4), we observed that the cytoskeleton of the remaining lamellocytes became irregular (Figure 4A,B) and that 38% of them had their nuclei condensed or fragmented compared to 2% after incubation in PBS, indicating a sharp drop in viability (Figure 4C).

### 3.3. Venom-Induced Ultrastructural Changes in Hemocytes

**Figure 5:**
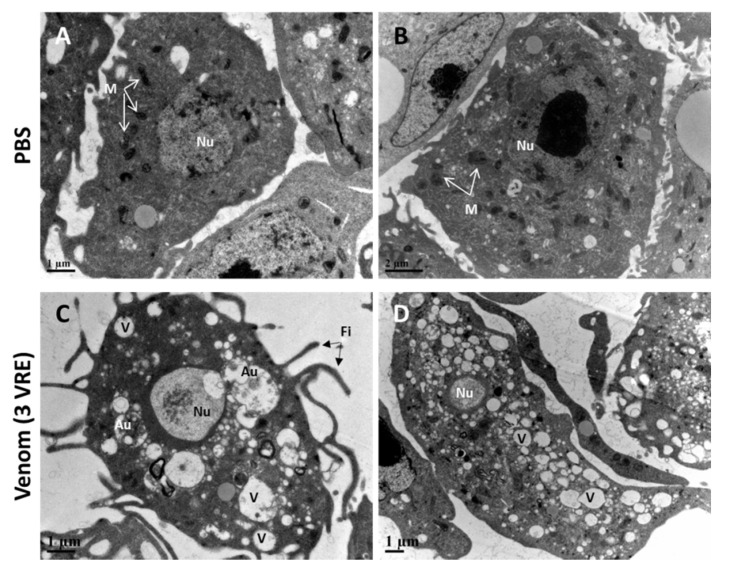
**Transmission electron microscopy (TEM) observation of hemocytes after venom treatment.** Ultrastructure of hemocytes collected from 3rd stage hop^Tum-l^ larvae which were incubated with PBS (**A**,**B**) or 3VRE (**C**,**D**). After 3 h of incubation with 3VRE, the shape of the nucleus (Nu) became very irregular, the vacuoles (V) were abundant in the cytoplasm and the mitochondria (M) disappeared in many hemocytes compared to the PBS condition. Plasmatocytes also had extended filopodia (Fi) and some autophagosomes-like structures (Au) were observed near their nucleus.

To uncover the mechanism of venom-induced changes in hemocytes, ultrastructural analyses were performed by transmission electron microscopy (TEM). PBS-treated hemocytes displayed large regular oval nuclei (Nu) with an euchromatic zone and regular shapes were observed for plasma membranes and mitochondria (M) of bot h plasmatocytes and lamellocytes (Figure 5A,B). In contrast, we observed various changes in the two types of hemocytes after 3 h incubation with 3 VRE (Figure 5C,D; see also Figure S6). There was in particular an extension of the filopodia (Fi) in the plasmatocytes (Figure 5C, Figure S6A,B) and an elongation of the lamellocytes (Figure 5D, Figure S6C,D) with, for bot h cell types, an extensive increase in cytoplasmic vacuolization (V) and a loss of recognizable mitochondria. Interestingly, we also observed autophagosomes-like (Au) structures in the cytoplasm near the nuclei of the plasmatocytes (Figure 5C, Figure S6A,B).

### 3.4. Venom-Induced Hemocytes Apoptosis

The changes observed in the properties, morphology and ultrastructure of hemocytes after incubation with the venom suggested the activation of an induced cell death mechanism. To test whether the type of cell death induced was linked to apoptosis, the TUNEL cell assay to analyze DNA damage [38] and the use of the caspase inhibitor Z-VAD-FMK (C_22_H_30_FN_3_O_7_) [19,39], have been carried out.

#### 3.4.1. TUNEL Staining

**Figure 6:**
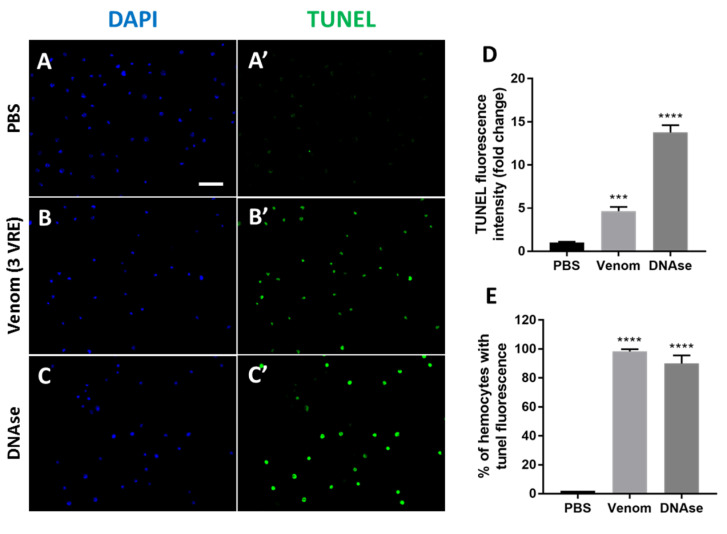
**Detection of apoptotic hemocytes by TUNEL assay**. Hemocytes collected from 3^rd^ stage hop^Tum-l^ larvae were incubated with PBS (**A**,**A’**), 3VRE for 3 h (**B**,**B’**) or 15 min with DNAse (positive control, **C**,**C’**). TUNEL labeling was visualized with green Alexa Fluor 488 (green) and nucleus with DAPI (blue). (**D**) The overall intensity of green fluorescence of different identical fields was measured. (**E**) Quantification of the percentage of hemocytes with TUNEL fluorescence. Mean ± S.D., (*n* = 3); ***: *p* < 0.001 and ****: *p* < 0.0001. Bar = 50 µm.

The nuclei of adherent hemocytes were stained with DAPI (4’,6-diamidino-2-phenylindole) and the occurrence of DNA breaks, indicating apoptosis, was investigated by the TUNEL tests after incubation with PBS as control or with venom (3VRE) (Figure 6A,B). While very few hemocytes (2%) were positive in the case of PBS, 98% were labeled in the venom condition (Figure 6A’,B’ respectively). This was confirmed by the 4.7-fold increase in the mean fluorescence intensity of the venom-treated hemocytes compared to the controls (Figure 6D). For the DNAse control treatment, 90% of hemocytes were TUNEL-positive (Figure 6C,C’) and the mean fluorescence intensity was increased 15-fold compared to that observed after treatment with PBS (Figure 6D).

#### 3.4.2. Caspases Involvement

Different pathways could be involved in apoptosis of *Drosophila* cells, several involving activation of caspases. To test the possible role of caspases in apoptosis of hemocytes, the hemocytes were exposed to crude venom (4.5 VRE) in the presence or absence of the caspase inhibitor Z-VAD-FMK and their morphological changes were observed (Figure S7). After 1 h, only 22% of the plasmatocytes had aggregated actin spots instead of 27% under the venom condition (Figure S7C) and the number of lamellocytes on the slide increased from 10% to 19% in the presence of the inhibitor (Figure S7D). Although the inhibitory effect was not significant for the modifications of the plasmatocytes, it was significant for the adhesiveness of the lamellocytes.

## 4. Discussion

In this study, we evidence a significant reduction in the number of hemocytes of *D. melanogaster* pupae after parasitism by the ectoparasitoid *P. vindemiae*. In addition, the in vitro incubation of hemocytes with crude venom reveals the alterations it induces in the morphology and ultrastructure of bot h the plasmatocytes and lamellocytes, eventually leading to cell death. This cell death of *D. melanogaster* hemocytes resembles apoptosis with reduction of cell volume, chromatin condensation and nucleus fragmentation [38]. We also observed the occurrence of a certain level of autophagy in the plasmatocytes, perhaps because they are phagocytic cells, as well as a large striking vacuolization and loss of mitochondria in bot h types of hemocytes, which could be also involved in cell apoptosis [40]. Although not definitive, our data also suggest that the caspase pathway may be partly involved in this process. We have tested different commercial antibodies against activated human caspase-3 but the only one which provided interesting results (an increased number of labeled cells after the venom treatment) has yet to be validated for *Drosophila* (Wan, personal observation). More experiments are thus required to decipher which caspase(s) is/are involved and then deduce what is the exact activation mechanism [41]. At least, the only partial effect of the caspases inhibitor suggests that there may be different types of venom-induced cell death mechanisms occurring simultaneously or sequentially. Since there are several pathways to apoptosis possibly induced by a variety of events triggered by cell surface receptors or others [42], further studies will be necessary to conclude clearly on the different mechanism and timing.

Based on this and on our previous observation [2] it would be wort h testing whether apoptosis could be a general effect on pupal tissues after envenomation. Indeed, our data indicate that different tissues, including the dorsal vessel, eyes and muscles, are strongly affected and we have also observed a dramatic increase in the number of apoptotic cells in the brain of an envenomated host over time after envenomation (Wan, personal observation). Venom or PDV-induced cell death has already been reported for several parasitic wasp species [5,12,19]. In other ectoparasitoids belonging to Pteromalidae, the venom performs similar functions. A sharp reduction in the number of *Sarcophaga bullata* plasmatocytes was observed at the preliminary stage after parasitism by *N. vitripennis* which was attributed to cell death and their ability to spread when cultured in vitro was rapidly lost [17]. *In vitro*, the *N. vitripennis* venom induced the rounding, swelling and eventually the death of different insect cell lines in a dose-dependent manner, suggesting a more or less general mechanism of action [43]. Cultures *T. ni* cells (BTI-TN-5B1) displayed indentations in their nuclear membrane, enlarged nucleoli and extensive vacuolization throughout the cytoplasm, cellular changes consistent with induced apoptosis [19]. However, *in vivo*, the venom of *N. vitripennis* induced morphological alteration and apoptosis in the brain cells of *S. bullata* but without any morphological or ultrastructural disturbance in fat body tissues [44]. The venom of *Pteromalus puparum*, an ectoparasite from the same family as *P. vindemiae*, caused a remarkable decrease in the percentage of plasmatocytes of its host *Pieris rapae* but the proportion of granular cells (another type of hemocyte) increased after parasitism, suggesting a more specific mechanism [45]. Therefore, the venom of pteromalids can have a general or a more or less specific effect depending on the conditions, the concentration and certainly the host species. In any case, the immune cells appear as one of the targets of the venom of ectoparasitoids, certainly with the aim of protecting the developing larvae.

Our recent study showed that the venom of *P. vindemiae* is a complex mixture of at least 64 proteins [28], some of which can potentially be cytotoxic and capable of inducing apoptosis. An example is calreticulin which is involved in cytotoxic processes [46] and also present in the venom of *N. vitripennis* [8,47] and *P. puparum* [48]. Among the other venom proteins of *P. vindemiae*, a gamma-glutamyltranspeptidase (GGT) has been identified. In *Aphidius ervi*, a GGT is the main protein of the venom and it seems to induce apoptosis of the host ovarioles perhaps by altering the metabolism of glutathione and therefore the oxidative stress [49]. Metalloproteases (MPs) are also known to participate in apoptosis [50] and there is one in the venom of *P. vindemiae*. A recombinant MP from the venom of *Eulophus pennicornis* was toxic to its host and probably manipulated the host development [8,51]. Moreover, MPs are widely distributed in the venoms of snakes and spiders as well, playing central roles in immune regulation and apoptosis-related processes [52,53]. At last, an endonuclease-like venom protein was also found, displaying a high identity with one of *N. vitripennis* [18], its function being possibly the induction of programmed cell death [54].

Overall, our findings demonstrate that the venom of *P. vindemiae* induces cell apoptosis in the *Drosophila* host, including that of different immune cells, likely as a protection strategy. The exact effectors involved and the mechanism/pathway are still unclear but the use of different *Drosophila* mutants as indicated here, will be helpful in identifying them as well as to detect the most sensitive tissues. In addition, being able to use larval hemocytes from *D. melanogaster* ex-vivo, which is much easier than using pupal tissues, will facilitate this molecular task.

## 5. Conclusions

The cellular immune interactions between parasitoids and their host insects are still central to invertebrate immunology, especially in *Drosophila*/parasitoids models. In this investigation, the aim was to excavate the mechanism by which the venom of *P. vindemiae* regulates the death of the hemocytes of the host *D. melanogaster*. The most significant finding from this study was that *P. vindemiae* venom causes a significant decrease in number of host plasmatocytes and lamellocytes by inducing cytoskeleton rearrangement, nucleus fragmentation and apoptosis. This investigation complements our earlier studies. Finally, an issue that was not addressed here was whether the effects were induced by specific components. Thus, considerably more work will need to be done to identify the effective venom components. In conclusion, the findings of this study may greatly contribute to the practical application of *P. vindemiae* on the control of *Drosophila*.

## Supplementary Materials

The following are available online at https://www.mdpi.com/2075-4450/11/6/363/s1, Figure S1: Number of hemocytes counted on the hemocytometer 10 h after parasitism by *P. vindemiae*. Figure S2: Observation of the plasmatocytes in the transgenic lines (Hml > GFP). Figure S3: Effects of *P. vindemiae* parasitism on the number of crystal cells (Lozenge > GFP). Figure S4: Observation of the lamellocytes in the transgenic lines (Atilla > GFP). Figure S5: Effect of the venom on the morphology of host hemocytes. Figure S6: TEM observation of the hemocytes after the venom treatment. Figure S7: Effect of the caspase inhibitor Z-VAD-FMK on the morphological changes of hemocytes. Table S1: Quantification of the numbers of hemocytes after incubation with the venom.
